# A Multi-Atlas Dynamic Connectivity Transformer Fused with 4D Spatiotemporal Modeling for Autism Spectrum Disorder Recognition

**DOI:** 10.3390/brainsci16040378

**Published:** 2026-03-30

**Authors:** Monan Wang, Jiujiang Guo, Xiaojing Guo

**Affiliations:** 1 School of Mechanical and Power Engineering, Harbin University of Science and Technology, Harbin 150080, China; 2 Second Clinical Medical College, Shanxi University of Chinese Medicine, Jinzhong 030619, China

**Keywords:** autism spectrum disorder (ASD), resting-state functional magnetic resonance imaging (rs-fMRI), dynamic functional connectivity (dFC), multi-scale, biomarker

## Abstract

**Highlights:**

**What are the main findings?**

- A dual-branch framework (MADCT-4D) is proposed to jointly model voxel-wise 4D spatiotemporal dynamics and temporally aligned multi-atlas dynamic functional connectivity for ASD recognition.
- The proposed framework demonstrates consistently superior performance on the ABIDE dataset compared with representative dynamic-connectivity and multi-view baselines.

**What are the implications of the main findings?**

- Temporally aligned fusion of 4D rs-fMRI representations and multi-atlas dFC provides a robust end-to-end solution for capturing transient brain coupling patterns.
- The framework provides interpretable cross-atlas biomarkers consistent with altered functional coupling in ASD, supporting explainable neuroimaging-based diagnosis.

**Abstract:**

**Background:** The recognition of autism spectrum disorder (ASD) has been a challenge due to the heterogeneity in symptoms and complex variations in brain function. Resting-state functional magnetic resonance imaging (rs-fMRI) has become instrumental in studying these disorders by accessing underlying abnormal neural activity and connectivity. Recently, deep learning approaches have shifted the analysis of brain networks by capturing spatiotemporal information from fMRI sequences. Nonetheless, most existing studies are limited by relying on a single representational scale, typically restricting analysis to either voxel-level spatiotemporal patterns or static connectivity matrices. Additionally, the dynamic reconfiguration of functional coupling and its variations across different anatomical parcellations are often ignored, which obscures neurobiologically meaningful dynamics. **Methods:** In this regard, we propose a multi-atlas dynamic connectivity transformer fused with 4D spatiotemporal modeling for ASD recognition (MADCT-4D). Specifically, the framework comprises two complementary branches. The 4D spatiotemporal branch encodes raw rs-fMRI volumes to learn hierarchical representations of evolving neural activity, while the dynamic-connectivity branch models time-resolved functional connectivity sequences constructed from multiple atlases, enabling the network to capture dynamic reconfiguration at the connectome level under different parcellation granularities. Moreover, we perform late fusion by combining the branch-specific decision scores with a learnable gate, allowing the model to adaptively weight voxel-level dynamics and multi-atlas connectivity evidence for each subject. **Results:** Extensive experiments on the publicly available ABIDE dataset demonstrate that the proposed method achieves 90.2% accuracy for ASD recognition, outperforming multiple competitive baselines. **Conclusions:** The proposed framework yields interpretable biomarkers based on learned dynamic connectivity patterns that are consistent with altered functional coupling in ASD.

## 1. Introduction

Autism spectrum disorder (ASD) is a highly prevalent neurodevelopmental condition that affects communication and behavior across the lifespan, and recent U.S. surveillance reporting has highlighted a continued increase in the estimated prevalence among children [1]. Because ASD diagnosis remains primarily symptom-based, objective neurobiological characterization is an active focus for improving mechanistic understanding and supporting more quantitative assessment [2]. Resting-state functional magnetic resonance imaging (rs-fMRI) provides a noninvasive window into large-scale brain organization, and functional connectivity (FC) analysis that summarizes statistical dependencies between regions has become a widely used approach for modeling ASD-related network-level alterations [3]. In parallel, machine learning methods built on connectivity representations have increasingly incorporated transformer-style architectures and multi-atlas inputs to enhance classification performance on public rs-fMRI resources such as the Autism Brain Imaging Data Exchange (ABIDE) [4].

In recent years, deep learning for rs-fMRI has increasingly moved from static summary connectomes toward explicitly modeling evolving spatiotemporal brain network organization, for example, via graph-evolution-based learning on dynamic brain networks [5]. In parallel, transformer-based self-supervised frameworks have been proposed to learn transferable 4D spatiotemporal representations directly from fMRI sequences, such as masked autoencoding with coupled spatial and temporal transformer modules [6]. For ASD recognition specifically, recent work has emphasized the use of hierarchical and multi-level feature extraction over functional brain networks for improving discrimination beyond single-scale representations [7]. Very recent trends further explore transformer-plus-graph hybrids, including self-supervised graph transformer designs, to jointly capture long-range dependencies and graph-structured priors for ASD classification from rs-fMRI-derived networks [8].

Despite the progress made, the neuroimaging characterization research of ASD has gradually shifted from static correlation to the depiction of dynamic coupling patterns. However, the existing rs-fMRI recognition process still often treats time-varying connectivity information as a “post-processing feature”, making it difficult to consistently retain its key temporal attributes related to behavioral phenotypes in an end-to-end model [9]. Meanwhile, although temporal models such as Transformers have been utilized for ASD detection and self-supervised pre-training to enhance the representational power of rs-fMRI, there is still a lack of a unified and alignable modeling and fusion mechanism between the learned 4D spatiotemporal representations and explicit dynamic connectivity representations, making it difficult to stably utilize dynamic coupling signals for discrimination [10]. Furthermore, the connectivity pattern is highly sensitive to brain region partitioning strategies. The connectivity features obtained from a single atlas may introduce bias or instability, limiting the model’s performance and interpretability to the choice of partitions. Therefore, there is an urgent need for an ASD recognition framework that can integrate dynamic connectivity information from multiple atlases and with 4D spatiotemporal representations [11].

To address these limitations, we propose MADCT-4D, a multi-atlas dynamic connectivity transformer fused with 4D spatiotemporal modeling for ASD recognition. MADCT-4D jointly learns (i) a 4D rs-fMRI spatiotemporal representation that preserves voxel-wise dynamics and (ii) explicit dynamic functional connectivity (dFC) sequences computed under multiple atlases, enabling complementary views of transient cross-regional coupling to be retained within a single end-to-end framework. Specifically, we introduce a multi-atlas dFC Transformer that encodes atlas-specific dFC streams and performs late fusion across atlases, while a 4D spatiotemporal backbone extracts temporally contextualized neuroimaging features from the rs-fMRI volumes. The two branches are integrated by a learnable fusion module that adaptively combines their predictive evidence at the logit level, allowing dynamic coupling cues to modulate decisions without sacrificing the fidelity of 4D spatiotemporal patterns. Extensive experiments on ABIDE demonstrate that MADCT-4D achieves competitive ASD recognition performance and provides interpretable multi-atlas dynamic connectivity signatures that are consistent with neurobiological hypotheses, supporting its potential as a robust computational framework for ASD identification.

## 2. Related Work

Recent deep learning pipelines for ASD recognition from rs-fMRI can be broadly categorized based on the representations they learn and the way temporal information is integrated into the model. Beyond neuroimaging-specific pipelines, recent work has also highlighted the broader role of neurotechnology-driven systems for detecting neurophysiological patterns associated with ASD. For example, Pergantis et al. provide a systematic review of assistive and emerging technologies for identifying stress and anxiety-related neurophysiological signals in children with autism, emphasizing the growing importance of objective technology-based biomarkers and computational analysis in ASD assessment [12].

A dominant line of work still relies on connectome-style features derived from regional time series, then applies machine learning or neural feature reduction before classification. For example, denoising autoencoder-style pipelines compress high-dimensional atlas-based FC features into low-dimensional latent variables that aim to retain diagnostic information while improving computational efficiency and interpretability [13]. In parallel, the field has increasingly shifted from static FC toward dFC to characterize time-varying coupling patterns. Methodological analyses highlight both the promise of dFC-driven learning and the practical pitfalls, including sensitivity to windowing, sampling, and interpretability of learned temporal dependence, which together complicate robust end-to-end modeling [9]. More recently, Transformer-based spatiotemporal modeling has emerged as a strong alternative to purely graph-based or correlation-based pipelines, because attention mechanisms can directly model long-range dependencies in rs-fMRI sequences. BrainWaveNet demonstrates that transformer-style architectures can learn discriminative representations on ABIDE-like settings by explicitly encoding temporal structure instead of collapsing time too early [14]. Related transformer designs for brain disorder diagnosis propose structured spatiotemporal aggregation and reorganization modules to capture both inter-regional structure and temporal dynamics, and report strong results on ASD benchmarks [15]. Several studies have developed similarity-aware multi-view fusion strategies to integrate complementary functional network representations and improve diagnostic separability [16]. Dynamic connectivity has been modeled with attention-based graph transformers that leverage two complementary connectivity views, aiming to capture time-varying inter-regional interactions better [17]. Outside the transformer family, residual graph convolutional architectures combined with explicit spatiotemporal feature extractors have been explored to encode temporal variation directly within graph learning pipelines [18]. Finally, population-graph formulations have gained traction by incorporating inter-subject relations and cross-network node-level prediction, enabling ASD inference to benefit from cohort structure alongside individual brain connectivity patterns [19]. METAFormer proposes a multi-atlas enhanced transformer that takes flattened connectivity matrices from multiple parcellations (e.g., Automated Anatomical Labeling (AAL), Craddock 200 (CC200), and Dosenbach 160 (Dos160)) and uses masked-value self-supervised pretraining to boost ASD classification performance on ABIDE-I [20]. Qiang et al. introduce a deep learning framework that models interactions among hierarchical functional brain networks, arguing that hierarchical organization carries discriminative ASD information and improves rs-fMRI-based identification on ABIDE-style evaluations [21]. Alves et al. build functional brain networks from rs-fMRI and apply machine-learning classification with network-level organization (rather than a single pairwise metric), achieving robust ASD vs. control discrimination and offering interpretable network findings [22]. Zhang et al. propose specificity-aware federated graph learning (SFGL) for rs-fMRI disorder identification, combining shared and personalized branches to handle multi-site heterogeneity while training without centralizing data, and demonstrating improved generalization in cross-site settings [23]. MADE-for-ASD presents a multi-atlas deep ensemble that integrates multiple parcellations via a weighted ensemble and incorporates demographic information to improve ASD diagnosis robustness on ABIDE-I, with reported gains over prior single-view baselines [24]. MCDGLN models ASD with dynamic functional connectivity from sliding windows and introduces task-specific connection masking/refinement to denoise and prune irrelevant edges, coupled with graph-based feature extraction and attention to improve ABIDE-I classification [25]. Finally, an important and increasingly active direction is multi-atlas or multi-view integration, motivated by the empirical observation that connectivity patterns and downstream predictions can be sensitive to the parcellation choice. Recent multi-atlas learning work proposes explicit mechanisms such as cross-atlas distillation or representation alignment to reduce atlas-induced instability and to consolidate complementary information across parcellations [26].

Despite this rapid progress, important gaps remain. First, temporal dynamics are still often treated as auxiliary cues rather than being preserved throughout end-to-end representation learning. Second, current transformer or graph models usually learn 4D spatiotemporal features and explicit dFC features in parallel but without a unified alignment mechanism, making dynamic coupling hard to exploit consistently. Third, multi-atlas fusion reduces parcellation sensitivity, yet dynamic connectivity cues can still be weakened by naive feature-level fusion.

## 3. Materials and Methods

### 3.1. Overview

**Figure 1:**
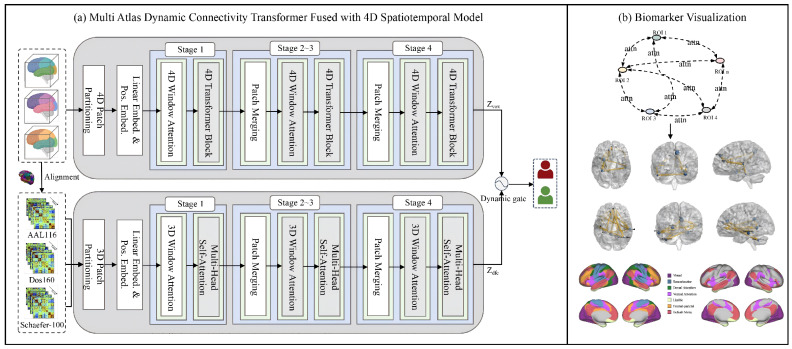
Overview of the proposed framework. (**a**) The overall architecture of the proposed model, which consists of a voxel-level 4D spatiotemporal branch and a multi-atlas dynamic functional connectivity (dFC) branch. The 4D branch encodes resting-state functional magnetic resonance imaging (rs-fMRI) volumes using a 4D Transformer to capture hierarchical spatiotemporal representations, while the dFC branch constructs time-resolved connectivity sequences based on multiple brain atlases, including Automated Anatomical Labeling (AAL116), Dosenbach 160 (Dos160), and Schaefer-100. The two branches are temporally aligned and fused via a learnable gating mechanism for autism spectrum disorder (ASD) recognition. (**b**) Visualization of the identified biomarkers, illustrating discriminative brain regions and connectivity patterns associated with ASD.

We propose a two-branch framework for ASD recognition that jointly leverages complementary information from raw 4D rs-fMRI and multi-atlas dFC, as shown in Figure 1. The first branch takes clipped 4D rs-fMRI volumes as input and learns spatiotemporal representations directly in an end-to-end manner, aiming to preserve both anatomical patterns and temporal dynamics. In parallel, the second branch encodes temporally aligned dFC sequences computed under multiple brain parcellation atlases, capturing transient coupling patterns that are not explicit in static connectivity features.

For downstream classification, the two branches are fused at the prediction level through a lightweight gating module, enabling the model to adaptively balance evidence from 4D spatiotemporal signals and dynamic connectivity cues.

During training, the dFC branch is activated only in the supervised ASD classification stage. In contrast, the pretraining stage refers to a self-supervised learning phase conducted solely on the rs-fMRI branch, where the 4D spatiotemporal backbone is trained without diagnostic labels to learn transferable spatiotemporal representations prior to downstream fine-tuning.

### 3.2. Dataset and Experimental Settings

Self-supervised pretraining of the 4D spatiotemporal backbone was conducted on raw rs-fMRI scans from ABIDE II, while all downstream ASD classification experiments and performance evaluations were performed exclusively on ABIDE I to ensure consistent benchmarking. For downstream experiments on ABIDE I, rs-fMRI data were obtained from the ABIDE Preprocessed Initiative and processed using the Configurable Pipeline for the Analysis of Connectomes (C-PAC; Child Mind Institute, New York, NY, USA), including motion correction, normalization to Montreal Neurological Institute (MNI) standard space, nuisance regression, temporal band-pass filtering (0.01–0.1 Hz), and spatial smoothing. Subjects with excessive head motion were excluded according to the standard quality-control criteria of the ABIDE Preprocessed Initiative. For the self-supervised pretraining stage, the 4D branch used rs-fMRI volumes derived from raw ABIDE II scans, which were preprocessed by us using the same pipeline as applied to the ABIDE I data (C-PAC pipeline including motion correction, normalization to MNI space, nuisance regression, temporal band-pass filtering, and spatial smoothing). No subjects from ABIDE I were used during the pretraining stage.

To construct dFC streams under complementary spatial granularities, we use three atlases: AAL116 [27], Schaefer-100 (7-network version) [28,29], and Dos160 [30]. Regional time series are obtained by averaging voxel-wise signals within regions of interest (ROIs), followed by sliding-window correlation.

For the rs-fMRI branch, each sample is represented as a 4D clip of shape 96×96×96×T with T=20 volumes (stride δ=1). For the dFC branch, we use a window length of 60 s and step size of 4 s (TR =2.0 s), and temporally align the dFC subsequence to the rs-fMRI clip.

To mitigate multi-site confounding, all experiments were conducted using subject-wise 5-fold cross-validation with joint stratification on diagnostic label (ASD vs. typically developing controls (TC)) and acquisition site (SITE_ID), ensuring balanced class and site distributions across folds.

Unless otherwise stated, we train using AdamW (learning rate $1\times 10^{-4}$, weight decay 0.01, batch size 16) and optimize cross-entropy loss. The proposed model contains approximately several million trainable parameters. To reduce overfitting risk, we apply weight decay regularization and monitor validation performance during training.

### 3.3. rs-fMRI and dFC Representation

For each subject, we represent the preprocessed rs-fMRI as a 4D blood-oxygen level-dependent (BOLD) volume $X\in R^{Z\times Y\times X\times T_{s}}$. To handle variable scan lengths, we adopt clip-based sampling by selecting a start index $t_{0}$ and extracting a clip of length *T* with optional within-clip stride δ. After symmetric padding or center-cropping, each clip is standardized to a fixed-size voxel sequence ${\tilde{x}}_{t_{0}}\in R^{96\times 96\times 96\times T}$, which is fed into the 4D encoder. This enables the voxel branch to learn spatiotemporal representations from raw BOLD dynamics with consistent input size.

In parallel, we explicitly represent time-varying inter-regional coupling using dFC. Given an atlas a∈{aal, sch, dos} with $N_{a}$ parcels, we first obtain atlas-level regional time series $R^{\left(a\right)}\in R^{T_{s}\times N_{a}}$ by averaging BOLD signals within each parcel. Then, we compute windowed connectivity using a sliding window of length *W* volumes and step size *S* volumes. For the *k*-th window $W_{k}$, we form the dFC matrix $C_{k}^{\left(a\right)}$:

(1) $$C_{k}^{\left(a\right)}=\mathrm{Corr}\;\left(R^{\left(a\right)}\left(W_{k}\right)\right)\in R^{N_{a}\times N_{a}},\;W_{k}=\left\{\left(k-1\right)S+1,\ldots ,\left(k-1\right)S+W\right\}$$

where Corr(·) denotes a correlation operator applied across regions within the current window. Stacking matrices over all windows yields a subject-level dFC sequence $C^{\left(a\right)}\in R^{L_{s}\times N_{a}\times N_{a}}$, with $L_{s}=\left\lfloor \frac{T_{s}-W}{S}\right\rfloor +1$.

A key requirement of our dual-branch design is temporal alignment between the voxel clip and the dFC subsequence. We therefore map the rs-fMRI clip start index $t_{0}$ to the corresponding dFC start window by

(2) $$k_{0}=\left\lfloor \frac{t_{0}}{S}\right\rfloor$$

and extract an aligned dFC subsequence beginning at $k_{0}$. To ensure that the dFC branch covers the same temporal span as the voxel clip, we set the required dFC length *L* either by a user-defined hyperparameter or by matching the temporal coverage:

(3) $$L=\left\lceil \frac{T\delta }{S}\right\rceil$$

When the available windows are insufficient near the end of the scan, we apply zero-padding to keep a fixed-length representation, which avoids variable-length batching and stabilizes optimization.

As a result, each training instance contains one voxel clip and three aligned dFC sequences (one per atlas),

(4) $$\left({\tilde{x}}_{t_{0}},\;D_{t_{0}}^{\left(\mathrm{aal}\right)},\;D_{t_{0}}^{\left(\mathrm{sch}\right)},\;D_{t_{0}}^{\left(\mathrm{dos}\right)}\right)$$

where $D_{t_{0}}^{\left(a\right)}\in R^{L\times N_{a}\times N_{a}}$ is the extracted (and, if needed, padded) dFC segment. This representation explicitly preserves two complementary views of rs-fMRI: (i) voxel-level 4D spatiotemporal dynamics captured directly from ${\tilde{x}}_{t_{0}}$, and (ii) multi-atlas time-varying coupling patterns summarized by ${\left\{D_{t_{0}}^{\left(a\right)}\right\}}_{a}$. The subsequent modeling stage leverages this alignment to fuse end-to-end learned 4D features with explicit dynamic connectivity evidence in a temporally consistent manner.

### 3.4. 4D Spatiotemporal Backbone for rs-fMRI

We employ a 4D spatiotemporal Transformer backbone to encode voxel-wise rs-fMRI clips into compact representations while preserving both spatial organization and temporal evolution. Given an input clip ${\tilde{x}}_{t_{0}}\in R^{96\times 96\times 96\times T}$, we first partition it into non-overlapping 4D patches using a 3D spatial patch size $\left(p_{z},p_{y},p_{x}\right)$ and a temporal patch size $p_{t}$. Let *N* denote the number of resulting tokens. Each patch is flattened and linearly projected to an embedding of dimension *D*, producing the token sequence

(5) $$H_{0}\;=\;\phi \;\left(\mathrm{Patchify}\;\left({\tilde{x}}_{t_{0}}\right)\right)\in R^{N\times D}$$

where ϕ(·) is a learnable linear projection. Position encodings are added to retain 4D location information, and the sequence is processed by *L* Transformer stages:

(6) $$H_{l}\;=\;B_{l}\;\left(H_{l-1}\right),\;l=1,\ldots ,L$$

where $B_{l}\left(\cdot \right)$ denotes a hierarchical 4D Transformer block (e.g., window-based self-attention with MLP and residual connections).

To reduce the quadratic cost of global attention, self-attention is computed within local 4D windows. For a window containing $n_{w}$ tokens, the windowed multi-head self-attention is

(7) $$\mathrm{Attn}\left(Q,K,V\right)\;=\;\mathrm{Softmax}\;\left(\frac{QK^{⊤}}{\sqrt{d}}\right)V$$

with $Q=HW_{Q}$, $K=HW_{K}$, and $V=HW_{V}$, where *d* is the per-head dimension. Window shifting across layers encourages cross-window interaction and allows information to propagate across the full spatiotemporal extent of the clip.

Finally, we aggregate the final token sequence into a clip-level representation. In our implementation, we apply token pooling (average pooling over tokens) to obtain

(8) $$g_{\mathrm{fmri}}\;=\;\mathrm{Pool}\;\left(H_{L}\right)\in R^{D}$$

which is subsequently used for downstream ASD classification and for fusion with the explicit multi-atlas dFC branch.

### 3.5. Multi-Atlas Dynamic Functional Connectivity Modeling

While the 4D voxel backbone learns spatiotemporal patterns directly from BOLD dynamics, we additionally introduce an explicit dFC pathway to model time-varying inter-regional coupling under multiple parcellation granularities. For each subject and atlas a∈{aal, sch, dos} with $N_{a}$ regions, we assume a precomputed dFC tensor $C^{\left(a\right)}\in R^{L\times N_{a}\times N_{a}}$, where *L* denotes the number of sliding windows, and each slice encodes window-specific coupling patterns.

Given a voxel clip starting at volume index $t_{0}$ and spanning ΔT volumes, we temporally align the dFC sequence by mapping volumes to window indices using the window step in volumes *s*:

(9) $$l_{0}=\left\lfloor \frac{t_{0}}{s}\right\rfloor ,\;L_{\mathrm{need}}=\left\lceil \frac{\Delta T}{s}\right\rceil$$

We then extract an aligned dFC subsequence ${\tilde{C}}^{\left(a\right)}\in R^{L_{\mathrm{need}}\times N_{a}\times N_{a}}$ (with zero-padding when $L<L_{\mathrm{need}}$) so that the connectivity stream is synchronized with the voxel clip.

To obtain a compact descriptor for each atlas, the aligned dFC subsequence is tokenized and processed by an atlas-specific Transformer encoder $E_{a}\left(\cdot \right)$, producing an atlas-level embedding

(10) $$g_{\mathrm{dfc}}^{\left(a\right)}=E_{a}\;\left({\tilde{C}}^{\left(a\right)}\right)\in R^{D_{d}}$$

Finally, we integrate information across parcellation granularities via a learnable fusion operator F(·) to form a unified multi-atlas dFC representation

(11) $$g_{\mathrm{dfc}}=F\;\left(g_{\mathrm{dfc}}^{\left(\mathrm{aal}\right)},\;g_{\mathrm{dfc}}^{\left(\mathrm{sch}\right)},\;g_{\mathrm{dfc}}^{\left(\mathrm{dos}\right)}\right)\in R^{D_{f}}$$

The resulting $g_{\mathrm{dfc}}$ is temporally aligned to the voxel clip and summarizes dynamic coupling information under multiple atlas views. It is subsequently integrated with the 4D voxel pathway through decision-level late fusion, enabling the final classifier to jointly exploit raw spatiotemporal dynamics and structured time-varying inter-regional interactions.

### 3.6. DFC Transformer Encoder

For each atlas a∈{aal, sch, dos}, the aligned dynamic connectivity sequence is ${\tilde{C}}^{\left(a\right)}\in R^{L\times N_{a}\times N_{a}}$, where *L* is the number of dFC windows and $N_{a}$ is the number of regions. We encode ${\tilde{C}}^{\left(a\right)}$ into an atlas-specific representation that preserves temporal evolution and inter-regional interaction patterns, while remaining scalable across different atlas resolutions.

We partition each connectivity frame into non-overlapping spatial patches of size P×P and keep the temporal index intact. Let $M_{a}={\left(N_{a}/P\right)}^{2}$ denote the number of spatial patches (assuming *P* divides $N_{a}$). We flatten each patch and linearly project it into a *D*-dimensional token:

(12) $$e_{t,m}^{\left(a\right)}\;=\;W^{\left(a\right)}\;\mathrm{vec}\;\left({\tilde{C}}_{t}^{\left(a\right)}\left[\Omega_{m}\right]\right)+b^{\left(a\right)},\;t=1,\ldots ,L,\;m=1,\ldots ,M_{a}$$

where $\Omega_{m}$ indexes the *m*-th spatial patch, and $W^{\left(a\right)}\in R^{D\times P^{2}}$.

All tokens are concatenated into a single sequence $E^{\left(a\right)}\in R^{(LM_{a})\times D}$ and augmented with learnable positional embeddings to retain temporal ordering and patch layout:

(13) $$Z_{0}^{\left(a\right)}\;=\;E^{\left(a\right)}+P^{\left(a\right)}$$

where $P^{\left(a\right)}\in R^{(LM_{a})\times D}$.

We apply a stack of Transformer encoder blocks. At block l, multi-head self-attention models long-range dependencies across tokens:

(14) $$\mathrm{Attn}\left(Q,K,V\right)\;=\;\mathrm{Softmax}\;\left(\frac{QK^{⊤}}{\sqrt{d}}\right)V$$

where $Q=Z_{l-1}^{\left(a\right)}W_{Q}$, $K=Z_{l-1}^{\left(a\right)}W_{K}$, and $V=Z_{l-1}^{\left(a\right)}W_{V}$, and *d* is the per-head dimension. This formulation allows the encoder to learn temporally varying coupling patterns and their structured co-activations across matrix subregions.

To obtain a fixed-length atlas representation, we aggregate the final-layer tokens by global average pooling:

(15) $$g_{\mathrm{dfc}}^{\left(a\right)}\;=\;\frac{1}{LM_{a}}\sum_{i=1}^{LM_{a}}Z_{L_{T}}^{\left(a\right)}\left[i\right]\;\in \;R^{D}$$

where $L_{T}$ is the number of Transformer layers. The result $g_{\mathrm{dfc}}^{\left(a\right)}$ is then used for multi-atlas fusion and downstream integration with the 4D rs-fMRI branch.

### 3.7. Fusion Strategy Between rs-fMRI and dFC Branches

We fuse the rs-fMRI voxel branch and the multi-atlas dFC branch at the decision level to avoid feature scale mismatch and to preserve the specialization of each pathway. Let $g_{\mathrm{vox}}\in R^{D_{v}}$ be the clip-level representation from the 4D spatiotemporal backbone, and let $g_{\mathrm{dfc}}\in R^{D_{f}}$ be the fused multi-atlas dFC representation. Two lightweight heads map them to logits:

(16) $$z_{\mathrm{vox}}=h_{\mathrm{vox}}\left(g_{\mathrm{vox}}\right),\;z_{\mathrm{dfc}}=h_{\mathrm{dfc}}\left(g_{\mathrm{dfc}}\right)$$

where $z_{\mathrm{vox}},z_{\mathrm{dfc}}\in R^{C}$ for *C* classes, with C=2 for ASD versus control.

To adaptively balance the two predictions, we introduce a learnable gate α∈(0,1) and compute the fused logits as

(17) $$\alpha =\sigma \left(\beta \right),\;z=\alpha \;z_{\mathrm{vox}}+\left(1-\alpha \right)\;z_{\mathrm{dfc}}$$

where β is a trainable scalar and σ(·) is the sigmoid function. This formulation allows the model to automatically assign a higher weight to the branch that provides more reliable evidence during training, while keeping fusion stable and parameter efficient.

The fused logits z are optimized end to end with the task loss. For multi-class classification, we apply a softmax cross-entropy objective

(18) $$L_{\mathrm{cls}}=-\sum_{c=1}^{C}y_{c}\log\left(\mathrm{softmax}{\left(z\right)}_{c}\right),$$

where y is the one-hot label vector.

In our implementation, the gate and both heads are trained jointly with the voxel and dFC encoders, enabling consistent gradients to flow into both branches and yielding a unified predictor that leverages raw 4D spatiotemporal dynamics together with explicit time-varying inter-regional coupling.

### 3.8. Confounding Factors and Stratified Subject-Wise Splitting

ABIDE I is a multi-site dataset and is known to contain substantial heterogeneity across acquisition site, sex distribution, age range, full-scale IQ (FIQ), and eye status at scan. To mitigate confounding-driven performance inflation, all experiments were conducted using subject-wise partitioning, ensuring that no clips from the same subject appear in more than one fold.

We employed stratified 5-fold cross-validation, where stratification was jointly performed on diagnostic label (ASD/TC) and acquisition site (SITE_ID). This procedure preserves an approximately similar ASD/TC ratio and site composition across folds. Within each training fold, validation data were selected from the training subjects for early stopping, without using any test-fold subjects.

To verify that major confounding variables were balanced across partitions, we summarize the fold-wise distributions of site, sex, age, FIQ (on subjects with valid FIQ values), and eye status (Table 1).

## 4. Results

To evaluate the effectiveness of the proposed framework, we compare MADCT-4D with several representative ASD recognition methods reported in the literature.

### 4.1. Comparative Experiments

We compare MADCT-4D with four representative baselines discussed in related work, including a dual-view dynamic-connectivity Transformer, a similarity-guided multi-view fusion model, a multi-atlas deep ensemble, and a masked dynamic graph learning network. For our method, we report results under 5-fold cross-validation on ABIDE, and present the mean and standard deviation across folds. To keep the comparison concise and consistent, we summarize Accuracy (Acc), Precision (Prec), F1-score (F1), and area under the receiver operating characteristic curve (AUC). If a metric is not reported in the original paper, we denote it as NR.

As shown in Table 2, MADCT-4D achieves the best overall performance, reaching 90.2±0.9% accuracy with high precision and F1-score and an AUC of 93.4±0.7% under 5-fold cross-validation. Compared with a representative dual-view dynamic-connectivity Transformer, MADCT-4D improves accuracy, indicating that temporally aligned multi-atlas dFC modeling provides more reliable diagnostic evidence when fused with voxel-level 4D representations. MADCT-4D also outperforms the similarity-guided multi-view fusion baseline in Acc and AUC, suggesting that explicitly encoding time-resolved connectivity streams contributes complementary information beyond static multi-view connectome fusion. Finally, MADCT-4D surpasses a strong multi-atlas ensemble baseline in terms of accuracy, supporting the benefit of integrating multi-atlas dynamic coupling with 4D spatiotemporal modeling in a unified end-to-end framework. Overall, these results indicate that integrating voxel-level 4D spatiotemporal modeling with multi-atlas dynamic functional connectivity enables the model to capture complementary neural patterns that are not fully represented by conventional single-view or static-connectivity-based approaches.

**Figure 2:**
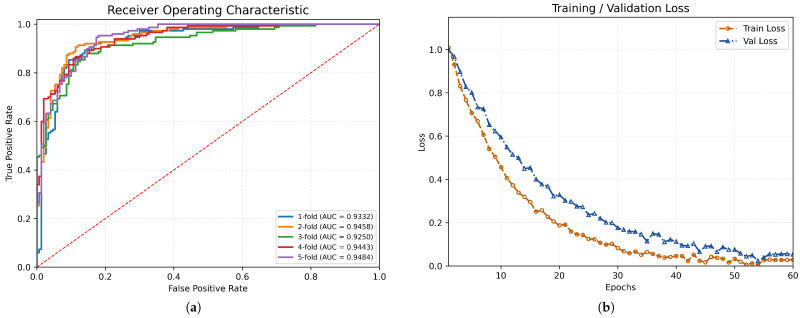
Cross-validation receiver operating characteristic (ROC) and optimization behavior of the proposed method on the ABIDE dataset. (**a**) ROC curves over 5-fold cross-validation on ABIDE. The AUCs for folds 1–5 are 0.9332, 0.9458, 0.9250, 0.9443, and 0.9484, respectively, indicating stable discriminative performance across splits. (**b**) Training and validation loss curves over epochs. Both curves decrease consistently and converge, suggesting stable optimization without obvious overfitting under our training setting.

Beyond the quantitative comparisons, we further visualize the fold-wise discriminative capability and the optimization behavior of the proposed model. Figure 2a shows the receiver operating characteristic (ROC) curves across 5-fold cross-validation, where the AUC values remain consistently high across folds, indicating stable discriminative performance under different train–test splits. Figure 2b plots the training and validation loss trajectories over epochs. Both curves decrease consistently and converge, suggesting stable optimization without obvious overfitting under our training setting. Together, these visual diagnostics provide complementary evidence that the proposed fusion framework not only improves final classification performance but also exhibits robust learning dynamics and consistent separability across folds.

### 4.2. Diagnosis Prediction from Site Identity Alone

To assess whether site-related heterogeneity alone could explain the observed ASD classification performance, we conducted an auxiliary binary classification analysis in which diagnostic labels (ASD vs. TC) were predicted using acquisition site identity (SITE_ID) alone. Specifically, SITE_ID was encoded as a one-hot vector and used as the sole input to a logistic regression classifier under the same subject-wise stratified 5-fold cross-validation protocol.

This auxiliary classifier achieved an accuracy of 64.62±0.10% and an AUC of 66.64±0.10%. These results suggest that acquisition site contains some diagnostic predictive signal in ABIDE I, which is consistent with the known multi-site heterogeneity of the dataset. However, the performance of this site-only diagnostic baseline remains substantially below that of MADCT-4D, indicating that the proposed model is not merely exploiting site identity to achieve its classification performance.

We emphasize that this analysis is not a multiclass site-classification experiment but rather a diagnosis-prediction sanity check designed to assess whether site identity alone could account for the observed classification performance.

### 4.3. Ablation Experiments

To quantify the contribution of each core module in our framework, we conduct ablation studies by constructing three controlled variants that remove one component at a time while keeping all other settings identical (5-fold cross-validation; metrics reported as mean ± std).

MADCT-4D-A: Removes the rs-fMRI 4D spatiotemporal Transformer branch and performs prediction using only the multi-atlas dFC pathway.

MADCT-4D-B: Removes the dFC Transformer encoder (including multi-atlas dFC encoding) and performs prediction using only the rs-fMRI 4D spatiotemporal branch.

MADCT-4D-C: Removes the dynamic gating module and replaces it with a fixed late-fusion rule, i.e., $z=\frac{1}{2}z_{\mathrm{vox}}+\frac{1}{2}z_{\mathrm{dfc}}$.

As shown in Table 3, MADCT-4D-A yields the largest performance drop, indicating that voxel-wise 4D spatiotemporal modeling provides essential discriminative evidence that cannot be fully recovered from connectivity streams alone. This is consistent with the role of the rs-fMRI Transformer in capturing hierarchical spatiotemporal patterns directly from BOLD dynamics.

MADCT-4D-B also degrades noticeably, demonstrating that the explicit dFC Transformer pathway contributes complementary information beyond voxel-level representation learning. In particular, modeling temporally resolved coupling under multiple atlases improves separability by injecting structured, time-varying inter-regional interaction cues that are not guaranteed to be preserved in a purely voxel-based encoder.

Finally, MADCT-4D-C produces a smaller but consistent decline, suggesting that the learnable gate plays an important role in stabilizing integration between the two decision sources. By adaptively weighting $z_{\mathrm{vox}}$ and $z_{\mathrm{dfc}}$, the gating module prevents suboptimal fixed blending and enables the model to exploit the more reliable branch evidence under different folds and subject-specific variability.

### 4.4. Biomarker

To elucidate the neurobiological significance underlying the model’s decision-making process and enhance diagnostic interpretability, we identify key biomarkers by quantifying the integrated contribution of each region of interest (ROI) in terms of feature representation and network interaction. Specifically, we analyze the model’s reliance on different brain regions for correctly classified ASD samples.

First, to assess the activity level of each brain region within the feature space, we extract the output feature vector $z_{b,r}$ corresponding to ROI *r* from the final layer of the Transformer. For a given subject *b*, the feature activation intensity of the region is defined by calculating the $L_{2}$ norm of its feature vector:

(19) $$F_{b,r}={∥z_{b,r}∥}_{2}$$

where $F_{b,r}$ reflects the magnitude of the contribution of ROI *r* to the final representation layer of the model.

Additionally, to evaluate the status of each region as an information hub within the whole-brain network, we utilize the attention matrix $A^{\left(b\right)}\in R^{N\times N}$ generated by the model. We calculate the sum of attention weights from all other nodes *j* to node *r* to quantify the degree to which the region is attended to by the global network:

(20) $$W_{b,r}=\sum_{j=1}^{N}A_{j,r}^{\left(b\right)}$$

Moreover, to derive a robust global importance score, we combine the aforementioned feature activation intensity with network attention. The final score for each ROI is computed as the average of the product of these two terms across all correctly classified samples:

(21) $$\mathrm{Score}\left(r\right)=\frac{1}{B}\sum_{b=1}^{B}\left(W_{b,r}\times F_{b,r}\right)$$

where *B* denotes the total number of correctly classified subjects. This scoring mechanism effectively filters for regions that exhibit both high-intensity feature expression and occupy a core recipient position in the brain network’s information flow, thereby establishing them as critical anatomical features for ASD recognition.

Furthermore, to elucidate the functional interaction patterns among these key brain regions, we extract the FC matrix based on the aforementioned attention matrix $A^{\left(b\right)}$. Specifically, the FC strength between ROI *i* and ROI *j* is computed by averaging the attention weights across all correctly classified samples:

(22) $${\mathrm{FC}}_{i,j}=\frac{1}{B}\sum_{b=1}^{B}A_{i,j}^{\left(b\right)}$$

Since attention weights are inherently directional, we apply a symmetrization operation to obtain an undirected functional connectivity representation:

(23) $${\mathrm{FC}}_{i,j}^{\mathrm{sym}}=\frac{1}{2}\left({\mathrm{FC}}_{i,j}+{\mathrm{FC}}_{j,i}\right)$$

Finally, based on the ROI importance scores derived above, we select the top ten brain regions with the highest contributions and extract the corresponding functional connectivity submatrix ${\mathrm{FC}}^{\mathrm{top}10}\in R^{10\times 10}$ for subsequent visualization and interpretation.

**Figure 3:**
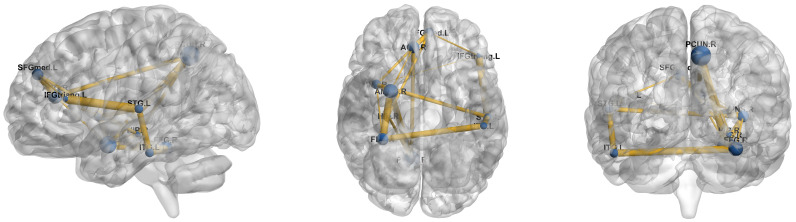
Top 10 brain connectivity patterns identified by our method in ASD recognition from AAL116.

**Figure 4:**
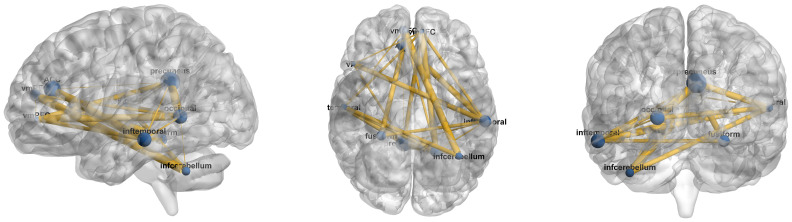
Top 10 brain connectivity patterns identified by our method in ASD recognition from Dos160.

**Figure 5:**
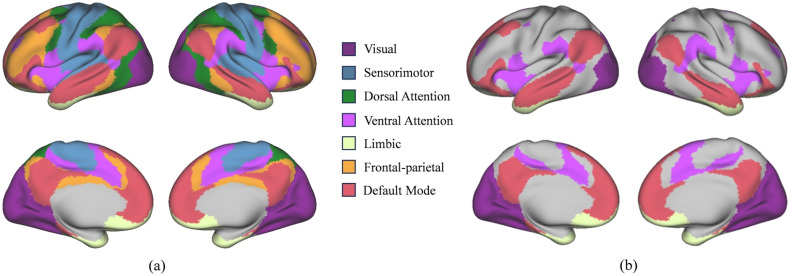
Top 10 brain connectivity patterns identified by our method in ASD recognition from Yeo 7. (**a**) The complete cortical surface mapping based on the Yeo 7 network functional parcellation. (**b**) The top 10 salient ROIs extracted from the Schaefer-100 parcellation. These regions, which demonstrated the highest discriminative importance for ASD diagnosis, are projected onto the cortical surface and color-coded according to their corresponding Yeo functional networks.

We conducted experiments on three distinct brain atlases: AAL116 [27], Dosenbach 160 (Dos160) [30], and Schaefer-100 [29]. By applying the same attribution analysis strategy across these atlases, which vary in spatial resolution and functional definition, we consistently identified anatomical regions and functional networks that play a pivotal role in ASD diagnosis. As illustrated in Figure 3 and Figure 4, the ten brain regions demonstrating the highest discriminative importance for ASD identification within the AAL116 and Dos160 atlases were visualized on the cortical surface using BrainNet Viewer (Beijing Normal University, Beijing, China) [31]. Table 4 and Table 5 provide a detailed list of these ROIs alongside their full anatomical nomenclature. As depicted in Figure 5, the top 10 salient ROIs identified from the Schaefer-100 parcellation were mapped onto the cortical surface. These regions were organized according to the seven canonical functional networks defined by Yeo et al. (2011), such as the Default Mode, Fronto-Parietal, and Visual networks. The cortical surface rendering for this visualization was performed using Connectome Workbench (Washington University School of Medicine, St. Louis, MO, USA) [32].

The cross-atlas analysis revealed a high degree of convergence. We consistently observed that the Precuneus and the medial prefrontal cortex (specifically vmPFC and SFGmed) exhibited high discriminative contribution in both the AAL116 and Dos160 atlases. These regions constitute the core nodes of the Default Mode Network (DMN) and spatially correspond to the areas color-coded in red (Default Mode) within the Yeo 7. Functional abnormalities in the DMN are widely recognized as a neuropathological hallmark of ASD, which are closely linked to the core deficits in self-referential processing and complex social cognition observed in patients [33,34,35,36]. Additionally, the anterior cingulate cortex (ACC), amygdala, and insula featured prominently among the top ten features in the AAL116 analysis, while the importance of the ACC was similarly highlighted in the Dos160 results. This finding is corroborated by the Yeo 7, where these regions align with the violet-coded Ventral Attention Network and the cream-coded Limbic Network. Functionally, these areas are primarily implicated in emotion regulation and salience monitoring [37,38]. Atypical activation patterns in the amygdala and insula are frequently associated with emotional dysregulation, anxiety symptoms, and diminished responsiveness to social stimuli commonly observed in individuals with ASD [39,40]. Furthermore, we observed strong discriminative influence from visual and temporal regions, specifically the fusiform gyrus (FFG) and inferior temporal gyrus (ITG) [41,42]. As a prominent region for face processing, altered functional connectivity in the fusiform gyrus may underlie the social deficits related to facial expression recognition and eye contact avoidance in ASD [43,44,45]. The cross-atlas consistency demonstrated by these results further validates the reliability of our proposed framework in identifying neuroanatomical biomarkers associated with ASD.

## 5. Discussion

This study introduces a dual-branch ASD recognition framework that fuses (i) voxel-wise 4D spatiotemporal modeling of rs-fMRI clips and (ii) temporally aligned multi-atlas dFC sequences. In this framework, the term “multi-view” refers to the complementary representations derived from voxel-level 4D spatiotemporal signals and atlas-level dynamic functional connectivity streams, while “multi-atlas” denotes the use of multiple brain parcellation schemes (AAL116, Schaefer-100, and Dos160) to construct connectivity representations at different spatial granularities. The main empirical finding is that jointly preserving these two complementary views yields consistently strong performance on ABIDE, with MADCT-4D achieving 90.2±0.9% accuracy and 93.4±0.7% AUC under 5-fold cross-validation, outperforming representative dynamic-connectivity Transformer and multi-view fusion baselines (Table 2). These results support the central hypothesis that ASD-related signatures may manifest both as voxel-level spatiotemporal patterns and as transient coupling reconfigurations that are not reliably retained when time-varying connectivity is treated as a post-hoc feature [9,17,20,24,25].

A key design choice is to compute dFC under multiple parcellations (AAL116, Schaefer-100, Dos160) and explicitly model each atlas stream before cross-atlas fusion. This design is motivated by parcellation sensitivity: the same underlying neural dynamics can project differently under different region definitions, affecting both the magnitude and topology of connectivity patterns. By learning atlas-specific dFC embeddings and fusing them with a learnable mechanism, the model can exploit complementary granularity cues and reduce reliance on any single atlas. The improvement over MADE-for-ASD (a strong multi-atlas ensemble baseline) suggests that multi-atlas gains are not merely due to ensembling across atlases, but also benefit from explicitly encoding the temporal evolution of connectivity within each atlas view [20,24,26].

The ablation study indicates that both branches contribute non-redundant information (Table 3). Removing the 4D voxel branch (MADCT-4D-A) causes the largest drop, implying that voxel-level spatiotemporal patterns contain discriminative signals beyond regional coupling summaries. Conversely, removing the dFC branch (MADCT-4D-B) also degrades performance, supporting the value of explicit time-varying coupling cues as an additional evidence stream. Importantly, these two representations are aligned in time: the voxel clip and dFC subsequence cover the same temporal span, which likely stabilizes fusion by preventing the model from combining temporally mismatched evidence.

We adopt logit-level late fusion with a lightweight learnable gate rather than concatenating heterogeneous features. The gain of MADCT-4D over the fixed-weight fusion variant (MADCT-4D-C) suggests that adaptive weighting is beneficial, likely because subject-level variability and site effects can make one branch more reliable than the other in different cases. This design also reduces the risk that high-dimensional dFC tokens dominate optimization simply due to scale or token count, a common issue in naive feature-level fusion. This decision is motivated by the relatively limited sample size of ABIDE and the heterogeneous nature of voxel-level and connectivity representations. Introducing feature-level cross-attention between these token types would substantially increase model complexity and may lead to unstable optimization or overfitting.

Beyond classification, the attribution analysis reveals convergent biomarkers across atlases, highlighting regions within the Default Mode Network (e.g., precuneus and medial prefrontal cortex) and salience/limbic-related nodes (e.g., ACC, amygdala, insula), consistent with prior evidence linking ASD to atypical DMN integration and altered salience/emotion regulation circuitry [33,34,37,38]. We also observe contributions from ventral visual and temporal regions (e.g., fusiform and inferior temporal cortex), aligning with widely reported ASD-related differences in social/face processing pathways [44,45]. Notably, these patterns emerge across AAL116, Dos160, and Schaefer/Yeo7 mappings, suggesting that the model is capturing robust network-level alterations rather than atlas-specific artifacts. We note that the attention-based importance scores should be interpreted as indicative patterns rather than causal explanations, since attention weights and feature magnitudes do not necessarily correspond to direct causal contributions of brain regions to the model’s decision.

Several limitations should be acknowledged. First, the dFC construction relies on sliding-window correlation, which is known to be sensitive to window length and step size and may blur fast transitions; although our approach mitigates this by end-to-end learning on the resulting sequences, the upstream estimator can still constrain what temporal structure is recoverable [9]. In this study, we adopt a 60-s window, which is widely used in rs-fMRI dynamic connectivity studies as a practical compromise between temporal sensitivity and statistical reliability. Nevertheless, sliding-window estimators inevitably provide only a coarse approximation of evolving connectivity states; future work may explore adaptive windowing or alternative dynamic connectivity estimators to better capture rapid coupling transitions. Second, ABIDE is multi-site and heterogeneous; while cross-validation indicates stability, further evaluation under strict site-held-out protocols would better characterize generalization under distribution shift. Third, our current fusion occurs at the decision level; while this improves stability, it may underutilize fine-grained cross-branch interactions (e.g., aligning voxel tokens with specific dFC edges or ROI pairs), which could further enhance interpretability and performance if designed carefully. Finally, the reported performance depends on preprocessing choices and atlas extraction details; systematic sensitivity analyses would strengthen reproducibility. Despite site-aware stratified partitioning and explicit quantification of site-related predictability, residual confounding effects may remain due to multi-site acquisition differences and incomplete phenotypic coverage. Future work will evaluate stricter site-generalization protocols (e.g., leave-one-site-out) and harmonization-aware modeling strategies. Although ABIDE II provides additional subjects, incorporating both releases would introduce further acquisition heterogeneity. Future work will evaluate joint training and cross-release generalization to assess robustness under increased multi-site variability.

Promising extensions include: (i) replacing sliding-window correlation with learnable or state-space-based dFC estimators to better capture rapid coupling changes; (ii) incorporating explicit cross-branch alignment modules (e.g., contrastive alignment between voxel tokens and atlas-level coupling tokens) to unify spatiotemporal and connectivity representations; (iii) adopting site-aware or federated training strategies to improve robustness without centralizing data, which is increasingly relevant for clinical translation [23]. More broadly, multi-atlas dynamic modeling provides a flexible foundation for integrating additional modalities (e.g., structural connectivity or phenotypic priors) and for probing ASD heterogeneity through subject-specific dynamic coupling signatures.

Overall, MADCT-4D demonstrates that temporally aligned fusion of 4D voxel dynamics and multi-atlas dFC streams improve ASD recognition while yielding interpretable biomarkers consistent with established neurobiological hypotheses. By explicitly modeling both evolving activity patterns and time-varying inter-regional coupling across parcellation granularities, the proposed framework offers a principled step toward more robust and explainable rs-fMRI-based ASD identification.

## 6. Conclusions

In this work, we proposed MADCT-4D, a two-branch ASD recognition framework that fuses voxel-wise 4D spatiotemporal modeling of rs-fMRI with temporally aligned multi-atlas dFC modeling. By preserving both evolving BOLD patterns and time-varying inter-regional coupling across complementary parcellations, MADCT-4D mitigates the limitations of single-scale or single-atlas pipelines and provides a more robust end-to-end representation for ASD identification. Experiments on the ABIDE dataset demonstrate that our method achieves strong and stable performance under 5-fold cross-validation, reaching 90.2±0.9% accuracy and 93.4±0.7% AUC, outperforming representative dynamic-connectivity and multi-view baselines. Ablation studies further confirm that both the 4D branch and the multi-atlas dFC branch contribute complementary diagnostic evidence, and that learnable decision-level gating improves fusion stability. Finally, cross-atlas biomarker analyses highlight consistent discriminative regions and network patterns (e.g., DMN- and salience/limbic-related circuitry), supporting the interpretability of the learned representations. These results indicate that multi-atlas dynamic connectivity fused with 4D spatiotemporal modeling is a promising and explainable computational framework for rs-fMRI-based ASD recognition.

Overall, the proposed MADCT-4D framework demonstrates that jointly modeling voxel-level spatiotemporal dynamics and multi-atlas dynamic connectivity can effectively capture complementary neural patterns for ASD recognition.

## Figures and Tables

**Table 1:** Distribution of major confounding variables across the 5 cross-validation test folds in the Autism Brain Imaging Data Exchange (ABIDE I), with subject-wise stratified by diagnosis and site. M/F: male/female. Eye status codes are from ABIDE phenotypic records (Open/Closed). Full-scale IQ (FIQ) is reported on the subset with valid values (FIQ >0).

Fold	*N*	ASD/TC	#Sites	Sex (M/F)	Age (Years)	FIQ *n*	FIQ	Eye (Open/Closed/NA)
1	223	107/116	20	194/29	17.6 ± 8.5	207	108 ± 15	147/76/0
2	223	107/116	20	190/33	16.8 ± 8.5	211	109 ± 15	160/63/0
3	222	107/115	20	183/39	17.4 ± 8.4	208	107 ± 16	155/67/0
4	222	109/113	20	187/35	16.8 ± 7.2	206	109 ± 15	154/68/0
5	222	109/113	20	194/28	16.7 ± 7.6	208	108 ± 15	149/73/0

**Table 2:** Performance comparison on ABIDE. Mean ± standard deviation is reported when provided by the original paper. NR denotes not reported. Performance values for competing methods are reported as published in the corresponding papers, since reimplementing all baselines under identical preprocessing pipelines and training protocols is beyond the scope of this study.

Method	Acc (%)	Prec (%)	F1 (%)	AUC (%)
Dynamic graph transformer [17]	74.6±1.6	76±7	NR	77.6±2.9
PC + SR + tHOFC [16]	79±4	79±4	81±4	83±4
MCDGLN [25]	73.3	73.0	69.7	NR
MADE-for-ASD [24]	88.7	NR	NR	NR
MADCT-4D (ours)	90.2±0.9	90.8±1.1	90.1±1.0	93.4±0.7

**Table 3:** Ablation results on ABIDE (5-fold cross-validation, mean ± std).

Method	ACC (%)	Precision (%)	F1 (%)	AUC (%)
MADCT-4D (full)	90.2±0.9	90.8±1.1	90.1±1.0	93.4±0.7
MADCT-4D-A	85.1±1.6	85.5±1.7	85.0±1.6	90.2±0.9
MADCT-4D-B	87.5±1.4	87.6±1.5	87.4±1.3	92.1±0.9
MADCT-4D-C	89.0±1.2	88.9±1.3	89.0±1.2	93.5±0.8

**Table 4:** Top 10 regions of interest (ROIs) identified in ASD recognition from AAL116.

Region	Abbr.
Precuneus	PCUN.R
Amygdala	AMYG.R
Anterior Cingulate Gyrus	ACG.R
Fusiform Gyrus	FFG.R
Superior Frontal Gyrus, medial	SFGmed.L
Insula	INS.R
Inferior Temporal Gyrus	ITG.L
Superior Temporal Gyrus	STG.L
Hippocampus	HIP.R
Inferior Frontal Gyrus, triangular part	IFGtriang.L

Note. L: Left Hemisphere; R: Right Hemisphere.

**Table 5:** Top 10 ROIs identified in ASD recognition from Dos160.

Region	Abbr.
Precuneus	precuneus
Anterior Cingulate Cortex	ACC
Occipital Cortex	occipital
Inferior Temporal Cortex	inftemporal
Ventromedial Prefrontal Cortex	vmPFC
Fusiform Gyrus	fusiform
Inferior Cerebellum	infcerebellum
Temporal Cortex	temporal
Ventral Frontal Cortex	vFC

## Data Availability

The datasets used and analyzed in this study are publicly available from the 1000 Functional Connectomes Project/INDI repository. The link to the dataset is http://fcon_1000.projects.nitrc.org/indi/abide/ (accessed on 25 March 2026).
